# Structural Determinants Underlying the Interplay Between Fatty Acid Binding and S-Nitrosylation of Human Serum Albumin

**DOI:** 10.3390/ijms27219687

**Published:** 2026-10-29

**Authors:** Anastasia A. Batalova, Kamila V. Samodurova, Mikhail A. Vovk, Daria A. Belinskaia, Richard O. Jenkins, Nikolay V. Goncharov

**Affiliations:** 1 Sechenov Institute of Evolutionary Physiology and Biochemistry, Russian Academy of Sciences, Pr. Torez 44, 194223 St. Petersburg, Russia; 2 St. Petersburg State University, 7/9 Universitetskaya Nab., 199034 St. Petersburg, Russia; 3 Leicester School of Allied Health Sciences, De Montfort University, The Gateway, Leicester LE1 9BH, UK; 4 Department of Biological Chemistry, St. Petersburg State Pediatric Medical University, Litovskaya St. 2, 194100 St. Petersburg, Russia

**Keywords:** albumin, nitrosylation, fatty acids, allosteric effects, gene expression, NMR spectroscopy, molecular modeling

## Abstract

Human serum albumin (HSA) is a multifunctional protein whose biological properties are determined not only by its amino acid sequence but also by a complex interplay of functional sites, post-translational modifications, and endogenous ligands. However, the structural mechanisms by which these regulatory factors influence the functional state of HSA remain insufficiently understood. The aim of this study was to investigate the structural basis of the interplay between HSA functional regions, using Cys34 S-nitrosylation and fatty acid (FA) binding as model regulatory processes. For this purpose, the structural features and conformational dynamics of FA-free and FA-loaded native and nitrosylated HSA, as well as their interactions with glutathione (GSH) and S-nitrosoglutathione (GSNO), were studied using molecular docking and molecular dynamics (MD) simulations. Changes in the overall structure of HSA after incubation with GSNO were assessed by ^1^H NMR spectroscopy. MD analysis revealed that FA binding alters the conformational dynamics of HSA and affects the structural environment of Cys34 and other functionally significant regions. These FA-induced effects were further modulated by S-nitrosylation, which influenced the conformational landscape of albumin in a manner dependent on its fatty acid-bound state. The similarity of the one-dimensional ^1^H NMR spectra of HSA before and after incubation with GSNO suggests the absence of pronounced changes in the overall protein structure upon interaction with the nitrosylating agent. The obtained data provide insights into how FA binding and S-nitrosylation shape the conformational properties of HSA through modulation of the structural environment of Cys34 and other functionally significant regions. The results highlight the importance of considering post-translational modifications and endogenous ligands as key determinants of HSA structure–function relationships.

## 1. Introduction

Human albumin (HSA) is the major protein fraction in blood plasma, accounting for over 50% of circulating proteins. HSA is vital for maintaining plasma oncotic pressure and regulating fluid balance between tissues and the vascular system. Furthermore, albumin acts as a universal carrier of its main endogenous ligand, fatty acids (FAs), as well as bilirubin, hormones, bile acids, metals, and a wide range of drugs, thereby maintaining systemic homeostasis and significantly mediating drug pharmacokinetics [1,2]. In clinical practice, HSA is used to treat severe burns, hemorrhagic shock, cirrhosis with ascites, hypoproteinemia, and related conditions [3]. The global demand for HSA exceeds 500 tons per year, significantly exceeding the capacity of natural plasma sources [4]. However, the role of albumin in the body is not limited to oncotic and transport functions. HSA has antioxidant and enzymatic properties that help maintain redox balance and promote the detoxification of certain xenobiotics [5,6]. Finally, it has been established over the years that HSA is capable of non-specifically interacting with certain enzymes and receptors expressed in the membranes of bloodstream and brain parenchyma cells [7,8,9].

Each of these functions of HSA is determined by a set of protein structural determinants, including low-molecular-weight ligand binding sites, redox-active centers, and surface regions that facilitate interaction with macromolecules. Despite the relative functional specialization of these centers, they are parts of a single dynamic system, which is the conformationally flexible HSA molecule. Therefore, changes in the structural characteristics of one center can affect the implementation of other albumin functions. More broadly, the functional state of HSA is determined not only by the expression of the *ALB* gene but also by the structural organization of the protein, its conformational dynamics, and post-translational modifications. Therefore, characterization of protein structure and dynamics provides an essential level of understanding of how genetic information is translated into biological function.

One of the important functions of HSA is the storage and transfer of nitric oxide (NO) through its covalent binding to the free thiol group of Cys34 to form S-nitrosoalbumin (HSA-NO) [10,11]. However, the physiological role of HSA-NO extends beyond the passive transport and storage of NO. Nitrosylation of albumin can modulate vascular responses by transferring the NO group to low-molecular-weight thiols or membrane-associated proteins [12,13]. HSA-NO can inhibit platelet aggregation, which helps prevent thrombus formation [14], and protects endothelial cells from damage in conditions such as ischemia–reperfusion injury and acute lung injury [15,16]. Preservation or targeted modification of this function for biomedical applications requires an understanding of the structural factors determining the reactivity of Cys34 and the stability of HSA-NO.

The formation of HSA-NO, like the nitrosylation of thiol groups in other proteins, can occur via several mechanisms. The most direct pathway is transnitrosylation involving low-molecular-weight S-nitrosothiols, such as S-nitrosoglutathione (GSNO) or S-nitrosocysteine (CysNO), which results in the transfer of the NO group to the Cys34 thiol [17,18]. The reactivity of the Cys34 thiol group is determined by its nucleophilicity and steric accessibility. These characteristics, in turn, depend on the microenvironment of the thiol group, the structure of which can change after albumin modification or the binding of low-molecular-weight substances to the ligand-binding sites of the protein. Thus, the reactivity of the thiol group can be influenced by fatty acid binding and glycation [19]. Similarly, the accessibility of the nitrosylated thiol group of Cys34 to low-molecular-weight thiols, and as a consequence, the stability of HSA-NO, may also depend on the occupancy of the ligand-binding sites of the protein.

Despite numerous studies devoted to allosteric effects in the albumin molecule, the molecular mechanisms underlying these processes remain poorly understood. The aim of this study was to identify the structural mechanisms underlying the interactions between functional sites in HSA, using Cys34 nitrosylation and FA binding as an example. To achieve this goal, we analyzed the structural and dynamic characteristics of native, nitrosylated, and FA-loaded albumin using molecular dynamics (MD) simulations and ^1^H NMR spectroscopy.

## 2. Results

### 2.1. Building of Three-Dimensional Models for Molecular Modeling

**Figure 1:**
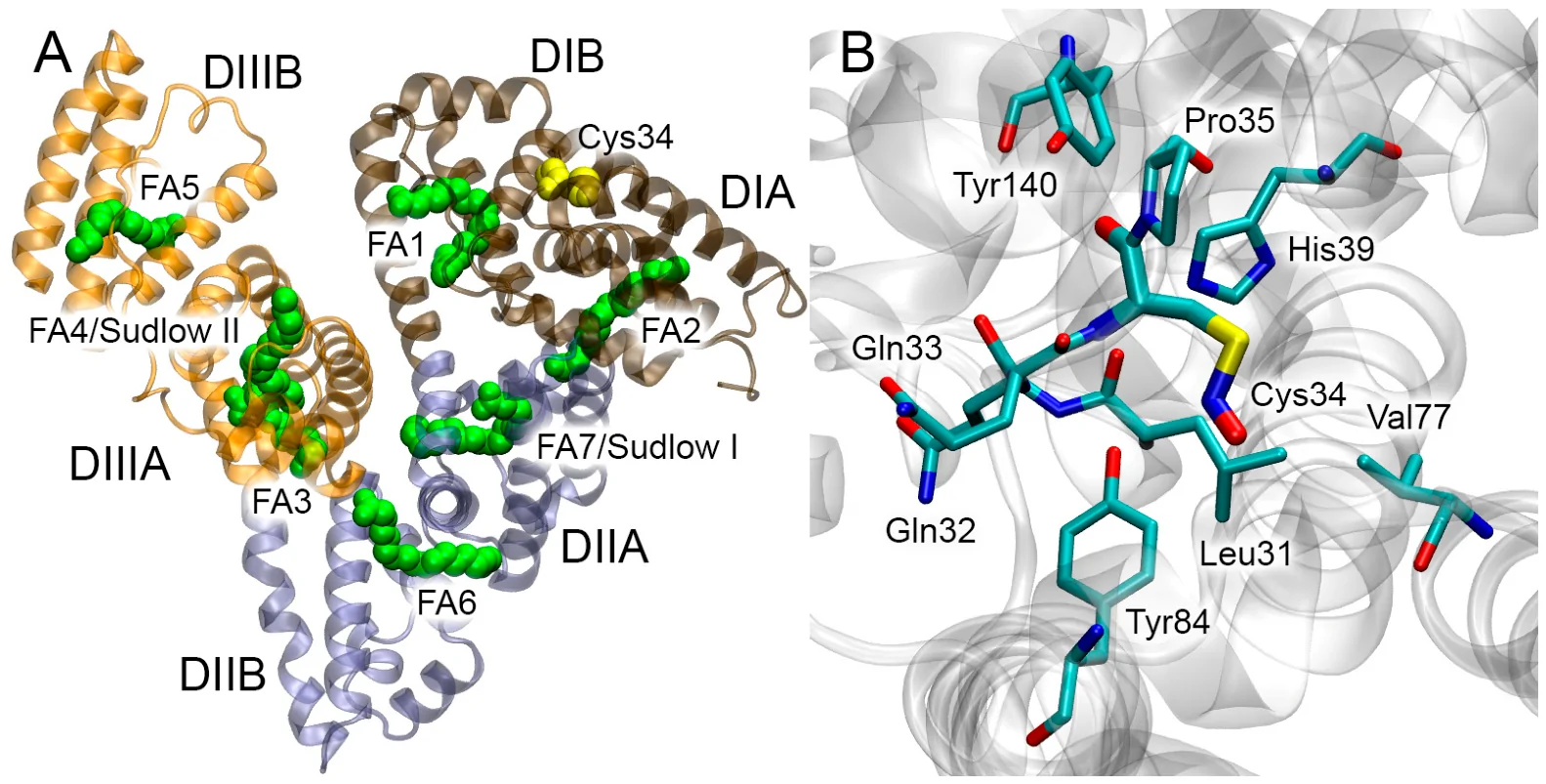
Topography of the human serum albumin (HSA) molecule using a model of nitrosylated and oleic acid (OLA) loaded albumin as an example. (**A**) domain structure, fatty acid binding sites (FA1–FA7), Sudlow I and II drug-binding sites, and the HSA redox site. Domains DI, DII, and DIII are shown as brown, silver, and orange ribbons, respectively, with their corresponding subdomains labeled as A and B. Oleic acid molecules bound in the FA sites are shown as green spheres, and the Cys34 residue is shown as a yellow sphere. (**B**) detailed structure of the amino acid environment of the nitrosylated Cys34 residue. The albumin backbone is represented as a gray ribbon, and atoms are colored by element: carbon in cyan, nitrogen in blue, oxygen in red, and sulfur in yellow.

The objects of in silico experiments were: native FA-free HSA; a complex of native HSA with 7 molecules of oleic acid (HSA-OLA) at fatty acid binding sites FA1-7; nitrosylated FA-free HSA (HSA-NO); and a complex of nitrosylated HSA with 7 molecules of oleic acid (HSA-OLA-NO). Crystal structures from the protein databank (PDB, [20]) were used to prepare the models. The 1AO6 structure [21] was used as a model of native HSA, and the 1GNI structure [22] was used as a model of HSA-OLA. Nitrosylated forms of albumin were constructed using the CHARMM-GUI web server (Lehigh University, Bethlehem, PA, USA) [23]. Figure 1A shows a spatial model of the HSA-OLA-NO complex, illustrating the arrangement of the domains, the FA1–7 fatty acid binding sites and the major HSA redox site Cys34, while Figure 1B shows the detailed structure of the amino acid environment of the nitrosylated Cys34. Site FA7 overlaps with drug site Sudlow I, and site FA4 overlaps with drug site Sudlow II.

### 2.2. Structural Characteristics of Various Modifications of Albumin According to Molecular Dynamics Simulation

**Figure 2:**
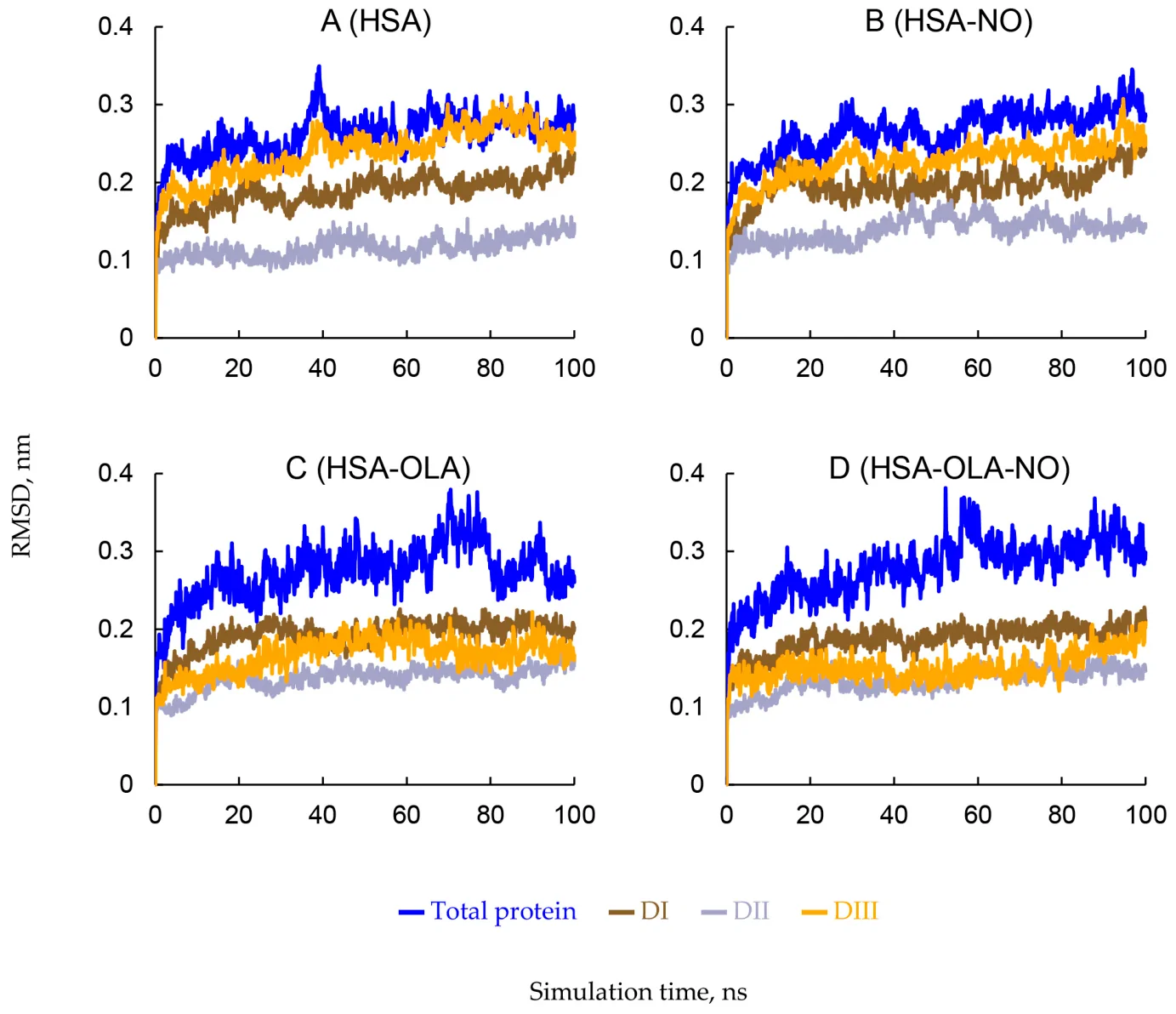
Time evolution of the root mean square deviations of Cα atoms of albumin relative to the starting position (RMSD), calculated for the entire albumin molecule (bright blue) and domains DI (brown), DII (silver) and DIII (orange). The calculation was performed for native FA-free albumin (HSA, (**A**)), nitrosylated FA-free HSA (HSA-NO, (**B**)), a complex of native HSA with 7 molecules of oleic acid (HSA-OLA, (**C**)) and a complex of nitrosylated HSA with 7 molecules of oleic acid (HSA-OLA-NO, (**D**)). Each plot represents the average dependence obtained from three MD replicas for each model.

After 3D-model preparation, we studied the conformational behavior of the HSA, HSA-NO, HSA-OLA, and HSA-OLA-NO models using MD simulations. Figure 2 shows the time evolution of the root mean square deviations (RMSD) of Cα atoms of each model relative to the initial position. The calculation was performed for the entire HSA molecule and for domains DI, DII and DIII separately. RMSD characterizes the stability of a protein molecule: if during the simulation the RMSD value reaches a plateau and does not increase further, this means that the system has reached a state of equilibrium and the structure is stable. For all models, the RMSD value of HSA gradually increases in the first 20 ns of the simulation (indicating gradual adaptation of the initial crystal structure to aqueous solution), then stabilizes at 0.3 nm (indicating that the protein has reached conformational equilibrium).

In the case of native FA-free albumin (the HSA model), the main contribution to the conformational changes in the protein molecule is made by conformational changes in the DIII domain; the DI domain is less conformationally mobile, and the conformation of the DII domain changes very little (Figure 2A). In the case of nitrosylated FA-free albumin (model HSA-NO), the distribution of domain flexibility is largely preserved, suggesting that Cys34 nitrosylation does not cause major changes in the conformational behavior of FA-free HSA (Figure 2B). In the case of OLA-loaded albumin (models HSA-OLA and HSA-OLA-NO), local conformational changes within individual domains are much less pronounced compared to the dynamics of the whole albumin molecule (Figure 2C,D). This suggests that the internal cores of the domains remain structurally stable during the simulation, whereas the global conformational changes in HSA are mainly associated with mutual spatial rearrangements (“hinge” motions) of the domains relative to each other.

**Figure 3:**
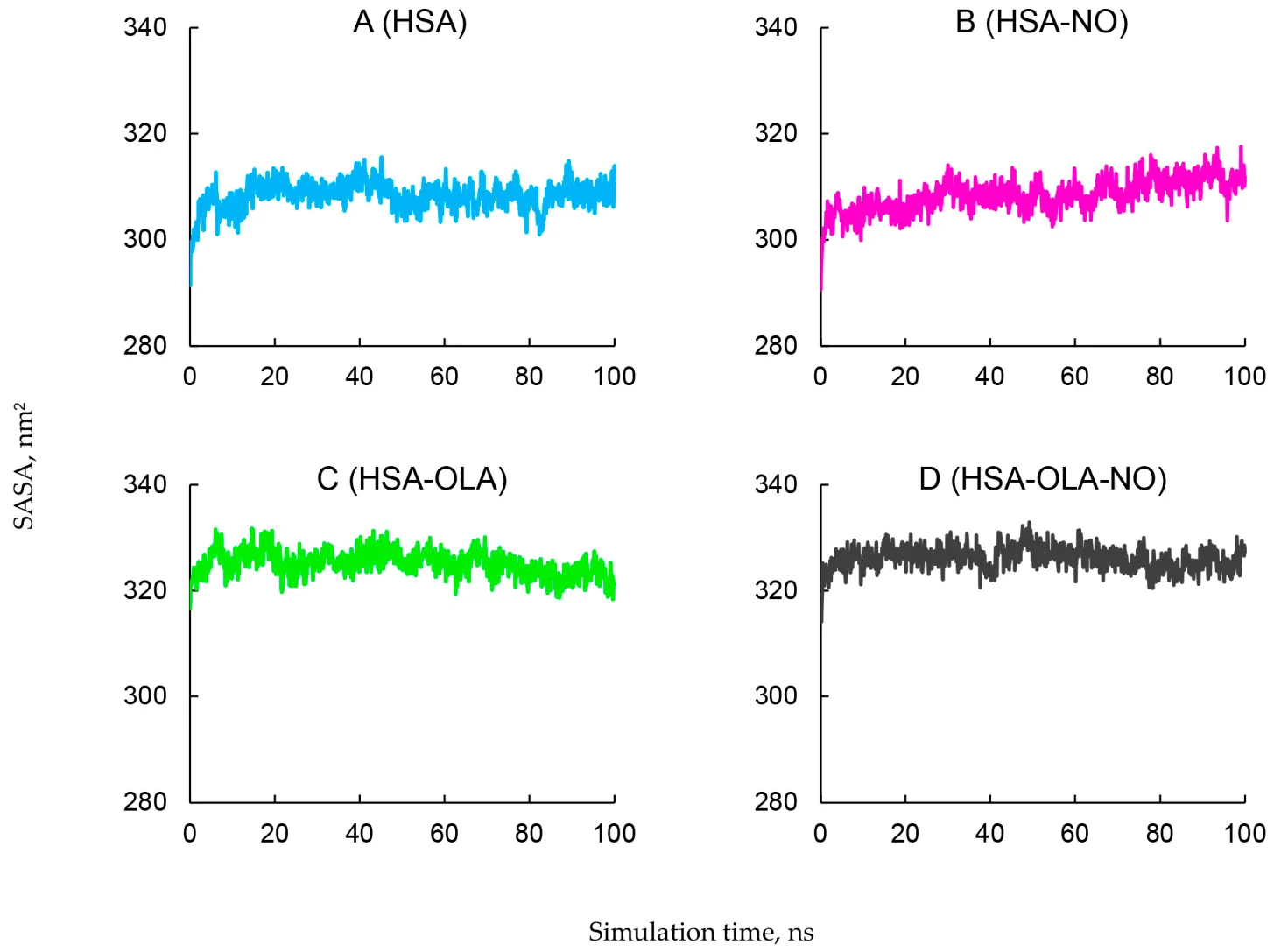
Time evolution of the solvent accessible surface area (SASA) of Cα atoms of albumin. The calculation was performed for native FA-free albumin (HSA, (**A**)), nitrosylated FA-free HSA (HSA-NO, (**B**)), a complex of native HSA with 7 molecules of oleic acid (HSA-OLA, (**C**)) and a complex of nitrosylated HSA with 7 molecules of oleic acid (HSA-OLA-NO, (**D**)). Each plot represents the average dependence obtained from three MD replicas for each model.

Figure 3 shows the time evolution of the solvent accessible surface area (SASA) of Cα atoms of albumin, which reflects how much the overall shape of the protein changes. An increase in SASA indicates the unfolding of the protein globule, in which hydrophobic regions, usually hidden inside, come to the surface and become accessible to solvent molecules. A decrease in SASA, on the contrary, indicates folding and compaction of the structure.

Since different crystal structures of albumin were used as the starting conformations of FA-free and OLA-loaded HSA, the SASA values are different for these models at the start of the simulation. As can be seen from Figure 3, the SASA value for FA-free albumin is lower than that for albumin complexed with OLA. During the simulation, SASA values for FA-free native and nitrosylated albumin (models HSA and HSA-NO, Figure 3A and Figure 3B, respectively) increased, with a more pronounced increase observed for HSA-NO. An increase in SASA may reflect several processes, including enhanced mobility of surface loops, increased exposure of previously buried regions, or changes in the relative arrangement of albumin domains. Comparison of SASA and RMSD profiles suggests that the increase in SASA observed for the HSA and HSA-NO models is mainly associated with increased flexibility of surface regions, particularly within the DI and DIII domains. In contrast, for the HSA-OLA model, after an initial increase associated with adaptation of the rigid crystal structure to the solvated environment, SASA values slightly decreased during the simulation (Figure 3C). This decrease may indicate stabilization of a more compact surface organization of the protein. In the HSA-OLA-NO model, the solvent-accessible surface area remained almost constant throughout the simulation (Figure 3D), suggesting preservation of a stable surface organization under the combined influence of oleic acid binding and Cys34 nitrosylation.

**Figure 4:**
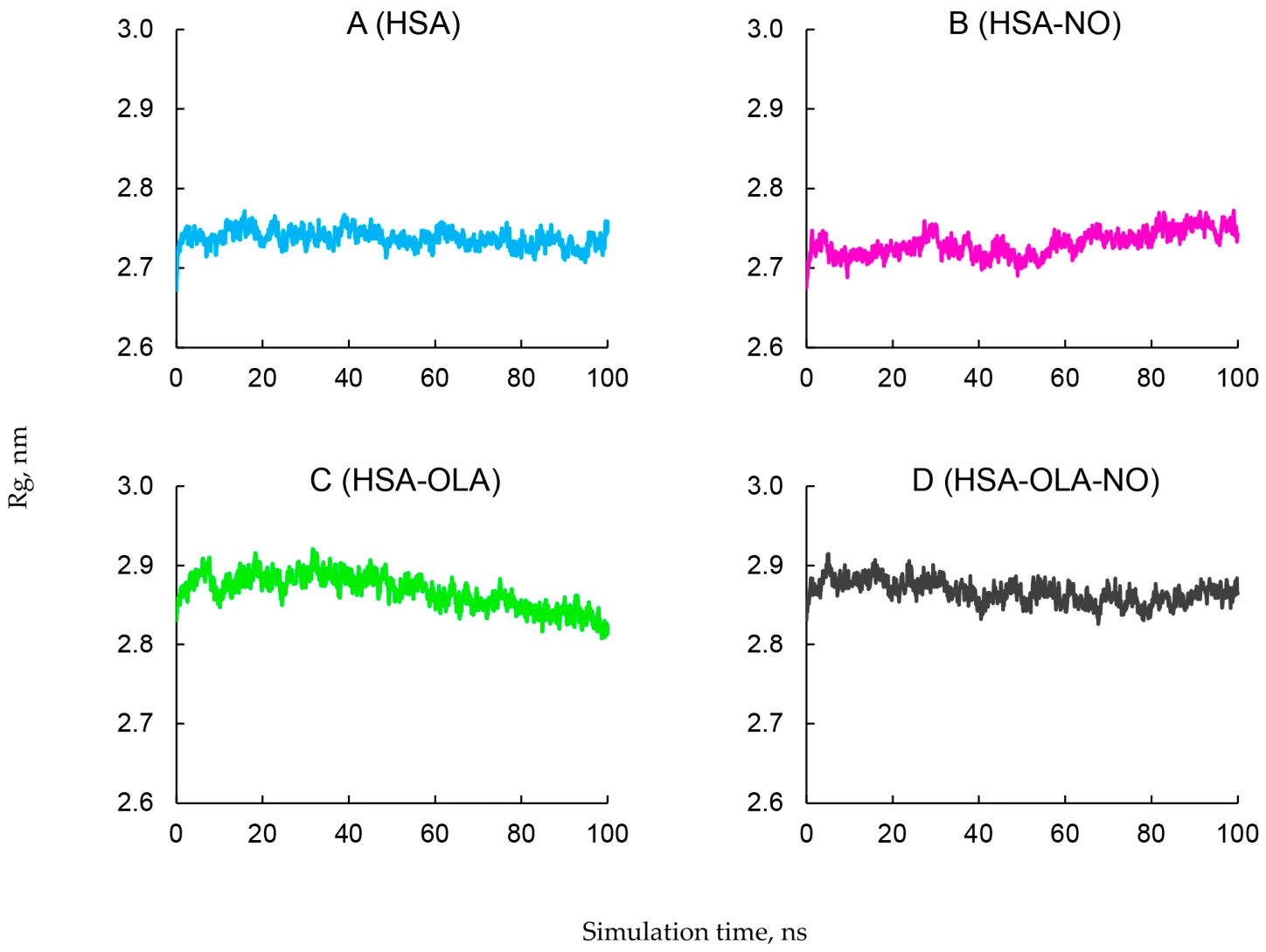
Time evolution of the radius of gyration (Rg) calculated for Cα atoms of albumin. The calculation was performed for native FA-free albumin (HSA, (**A**)), nitrosylated FA-free HSA (HSA-NO, (**B**)), a complex of native HSA with 7 molecules of oleic acid (HSA-OLA, (**C**)) and a complex of nitrosylated HSA with 7 molecules of oleic acid (HSA-OLA-NO, (**D**)). Each plot represents the average dependence obtained from three MD replicas for each model.

Similar trends are observed for the radius of gyration (Rg) (Figure 4). The Rg value characterizes the degree of protein compactness; an increase in this parameter during the simulation process means an increase in the degree of protein unfolding, and vice versa.

**Figure 5:**
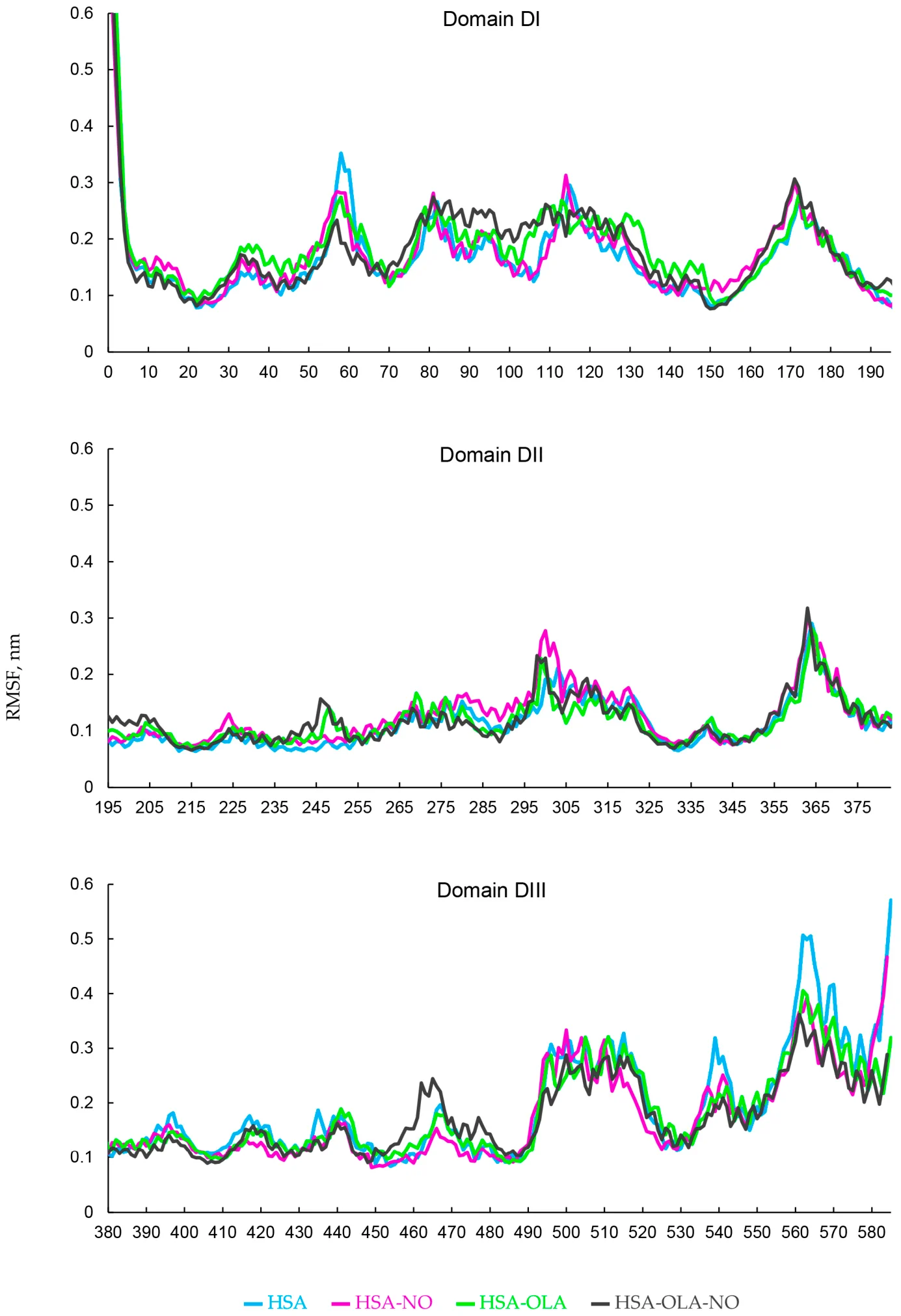
Values of the root mean square fluctuations (RMSF) of individual amino acids of albumin calculated for Cα atoms. The calculation was performed for native FA-free albumin (HSA, blue), nitrosylated FA-free HSA (HSA-NO, magenta), a complex of native HSA with 7 molecules of oleic acid (HSA-OLA, green) and a complex of nitrosylated HSA with 7 molecules of oleic acid (HSA-OLA-NO, gray). For clarity of the figure, each albumin domain (DI, DII and DIII) is represented by a separate fragment of the plot. Each plot represents the average dependence obtained from three MD replicas for each model.

Figure 5 shows the root mean square fluctuations (RMSF) of individual amino acids of albumin calculated for Cα atoms. The RMSF values characterize the local mobility of individual fragments of the protein molecule.

As Figure 5 shows, all the models studied exhibit similar distributions of local amino acid residue mobilities, with the most pronounced fluctuations observed in specific regions of domain DIII. In the FA-free albumin models (HSA and HSA-NO), a tendency toward higher RMSF values in domains DI and DIII was observed compared with the corresponding OLA-containing models, suggesting slightly increased local flexibility in these regions. This observation is consistent with the higher overall conformational variability reflected by the RMSD analysis (Figure 2). However, the differences in RMSF between the models were generally less pronounced than those observed in RMSD dynamics, suggesting that OLA binding primarily affects the global conformational behavior and relative motions of albumin domains, while preserving the overall pattern of local residue flexibility.

**Figure 6:**
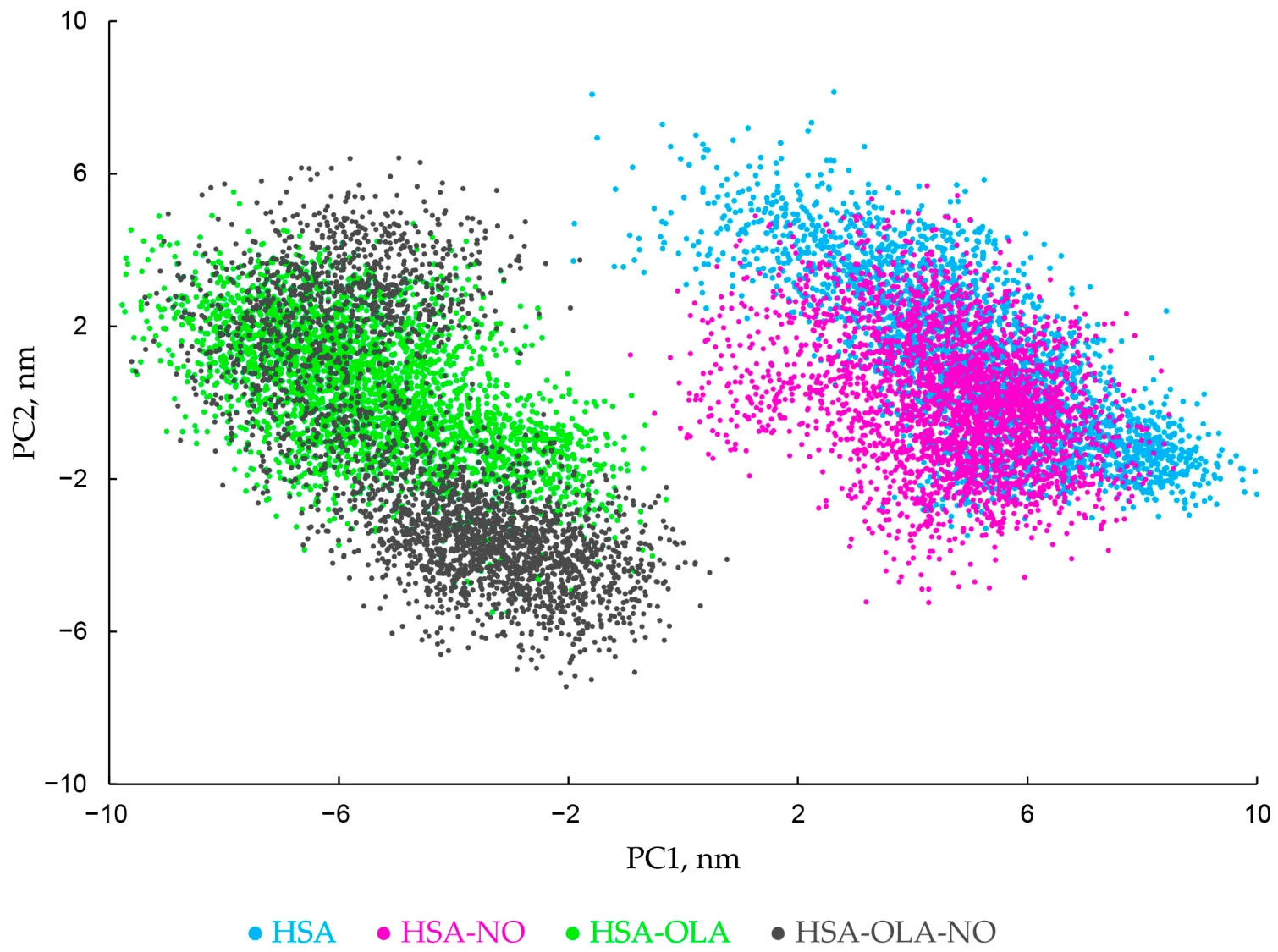
Principal component analysis (PCA) of the conformational dynamics of various albumin states: native FA-free albumin (HSA, blue), nitrosylated FA-free HSA (HSA-NO, purple), a complex of native HSA with 7 molecules of oleic acid (HSA-OLA, green), and a complex of nitrosylated HSA with 7 molecules of oleic acid (HSA-OLA-NO, grey). PCA was performed on the concatenated trajectory (4 models, 3 replicas each) encompassing all investigated HSA states to ensure the analysis of all systems within a shared conformational space.

To assess the influence of Cys34 S-nitrosylation and OLA binding on the global conformational dynamics of albumin, principal component analysis (PCA) was performed. To correctly compare different albumin states, PCA was performed on a combined trajectory that included all the models studied: HSA, HSA-NO, HSA-OLA, and HSA-OLA-NO. The analysis was carried out for the Cα-atoms of the protein, which made it possible to represent the conformational ensembles of all states in a single principal component space. The first two principal components (PC1 and PC2) described 63% of the total variance of atomic motions and were used to visualize the distribution of conformational states. The distribution of conformations in the PC1-PC2 space is shown in Figure 6. Each point corresponds to a separate frame of the MD trajectory. The location and extent of overlap between the conformational ensembles allow us to assess the influence of Cys34 modification and OLA binding on the dynamic behavior of albumin.

As shown in Figure 6, the most pronounced separation of conformational ensembles is associated with OLA binding: both FA-containing forms (HSA-OLA and HSA-OLA-NO) were shifted to the opposite region along the first principal component. This indicates that fatty acid binding is the primary factor driving the change in preferred conformational states of albumin. In contrast, FA-free forms (HSA and HSA-NO) exhibited significant overlap in their distributions, indicating that the global protein architecture is preserved after the modification of the Cys34 thiol group. Nonetheless, a slight redistribution of conformations along the second principal component suggests that S-nitrosylation can modulate the dynamic properties of the protein without inducing a large-scale rearrangement of its tertiary structure.

Interestingly, in the presence of OLA, the effect of S-nitrosylation manifested differently than in the case of the FA-free albumin. The conformational ensembles of HSA-OLA and HSA-OLA-NO maintained a close position along the PC1 axis, demonstrating the preservation of the primary motion mode driven by fatty acid binding. However, the HSA-OLA-NO complex was characterized by a broader distribution along PC2, reflecting an increase in the conformational heterogeneity of this state. This indicates that the Cys34 modification does not perturb the OLA-induced conformational transition but is capable of expanding the spectrum of accessible conformational states of the oleate-bound protein.

### 2.3. Conformational Analysis of Native and Nitrosylated Albumin Forms

**Figure 7:**
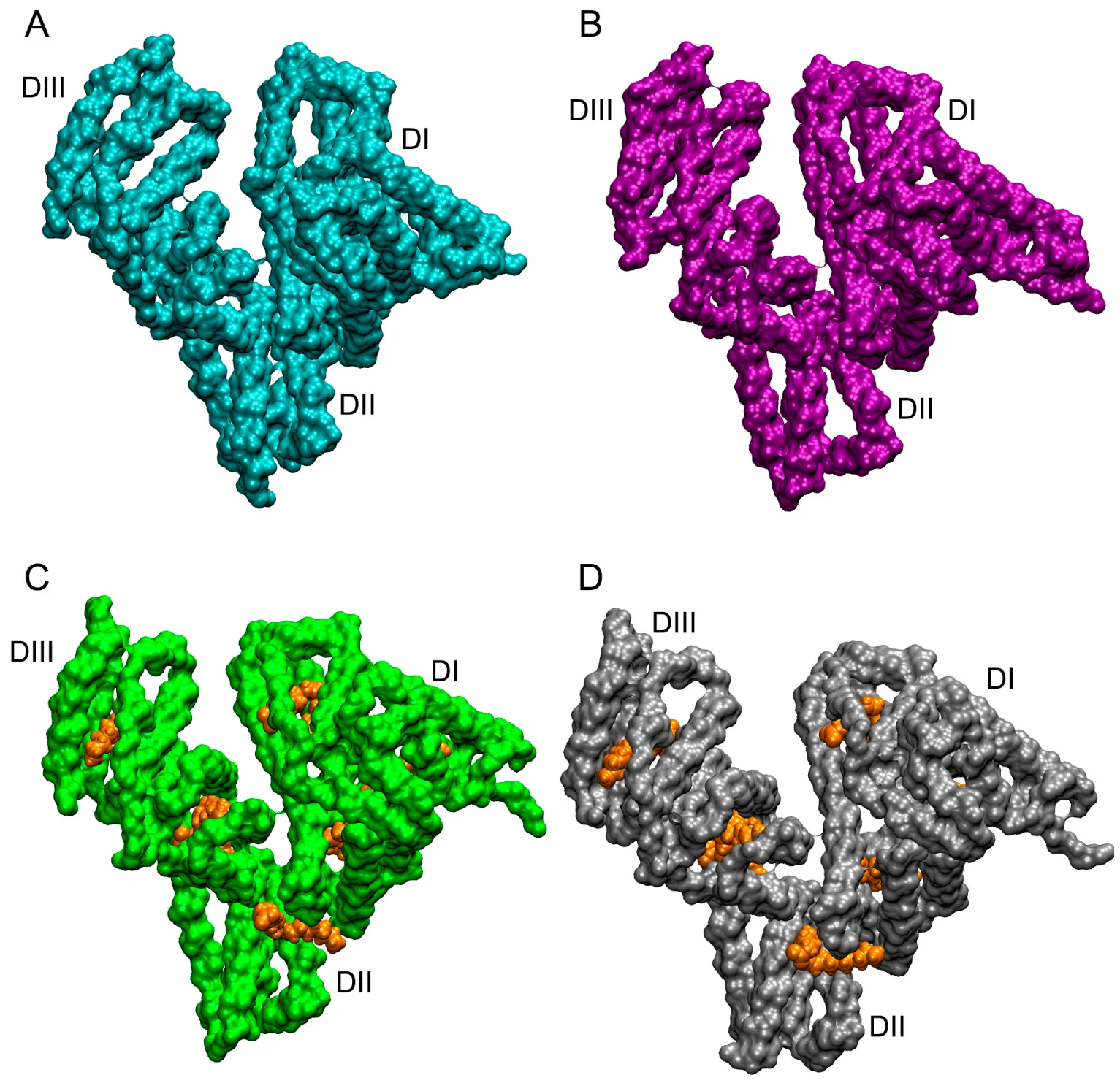
Conformation of various modifications of the albumin molecule: HSA (**A**), HSA-NO (**B**), HSA-OLA (**C**), and HSA-OLA-NO (**D**). The protein molecule is represented as a colored van der Waals surface of the backbone; the models HSA, HSA-NO, HSA-OLA, and HSA-OLA-NO correspond to blue, purple, green, and gray, respectively. OLA molecules are represented as orange spheres. Domains I, II, and III of albumin are designated by the symbols DI, DII, and DIII, respectively.

To analyze the conformational states of albumin, representative structures were selected as follows. For each MD trajectory, conformational clustering was performed using the software GROMACS (Stockholm University, Stockholm, Sweden) based on the 50–100 ns trajectory segment. Structures with an RMSD of Cα atoms below 1.5 Å were assigned to the same cluster. The most representative structure of the most populated cluster was then extracted using GROMACS. Thus, three representative structures were obtained for each model, one from each independent MD replica. Finally, the structure with the most favorable energy among the three selected conformations was chosen for each model. The resulting representative conformations are shown in Figure 7.

Comparison of the FA-free HSA forms (HSA vs. HSA-NO, Figure 7A,B) suggests that S-nitrosylation is accompanied by moderate changes in the overall shape of the protein, which is consistent with the trend toward increased SASA and Rg values of the HSA-NO model observed during MD simulations (Figure 3B and Figure 4B). However, the most pronounced rearrangement of the albumin conformation appears to be associated with oleate binding. Fatty acid binding is associated with a more extended conformation (HSA-OLA and HSA-OLA-NO, Figure 7C,D), which is reflected by the changes in SASA and Rg parameters observed during the MD trajectories (Figure 3C,D and Figure 4C,D). Notably, regardless of the fatty acid-bound state, S-nitrosylation was associated with a tendency toward a less compact conformational state of albumin. However, as indicated by the PCA (Figure 6), the structural basis of this effect differs depending on the presence of oleate: in FA-free albumin, S-nitrosylation is associated with moderate shifts in the overall conformational state, whereas in OLA-loaded albumin it affects the distribution of conformational states within the ensemble. The visual comparison of representative conformations (Figure 7) is consistent with the MD trajectory analyses, including RMSD, Rg, and PCA (Figure 2, Figure 4 and Figure 6). Together, these complementary approaches suggest that the conformational effects of S-nitrosylation depend on the ligand-binding state of HSA.

### 2.4. Conformational Analysis of the Redox Site of Native and Nitrosylated Forms of Albumin

**Figure 8:**
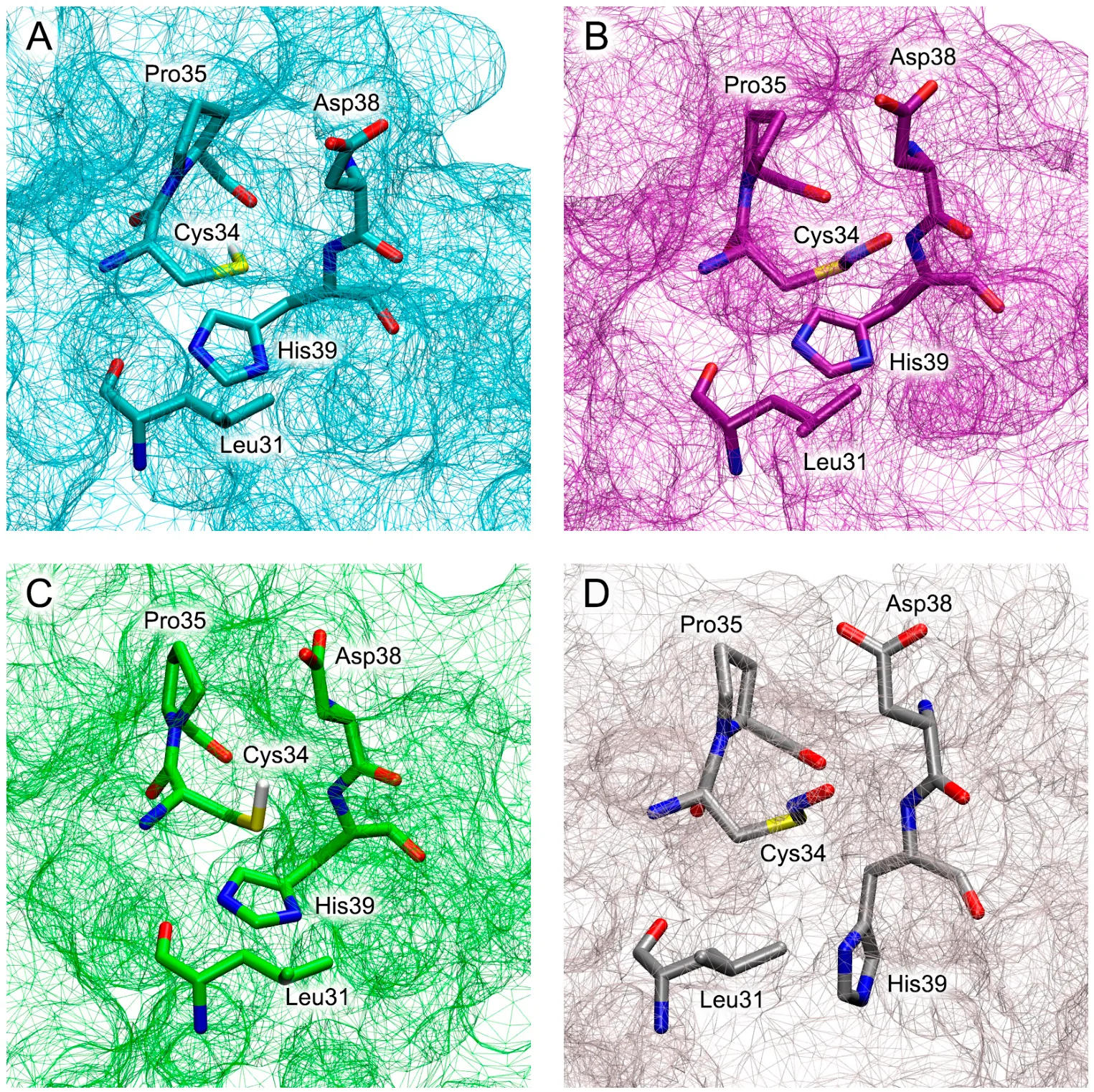
Conformation of the redox site of various albumin modifications: HSA (**A**), HSA-NO (**B**), HSA-OLA (**C**), and HSA-OLA-NO (**D**). The amino acids of the redox site are shown as sticks, while the remaining amino acids are represented as a colored surface; the HSA, HSA-NO, HSA-OLA and HSA-OLA-NO models are colored blue, purple, green and gray, respectively. Hydrogen atoms are not shown for clarity, except for the hydrogen atom of the free thiol group of Cys34.

In the next step, we analyzed how the conformation of the albumin redox site depends on nitrosylation and fatty acid loading of the protein (Figure 8). The same albumin structures used in the previous section were used for this analysis.

As shown in Figure 8, in the HSA model, the Cys34 thiol group is buried within the protein globule and has limited accessibility for interaction with nitrosylating agents (Figure 8A). S-nitrosylation of FA-free HSA induces changes in the redox site (Figure 8B). The nitrosylated Cys34 loses its interaction with the neighboring His39 residue; however, the sulfur atom of the SNO group at Cys34 remains buried within the protein globule. OLA binding also induces changes in the conformation of the redox site (Figure 8C). Notably, in the HSA-OLA model, the Cys34 thiol group appears to be the most accessible for transnitrosylation. S-nitrosylation of OLA-loaded albumin affects the conformation of the redox site (Figure 8D). In the HSA-OLA-NO model, the approach of Cys34 and Leu42 creates steric hindrance that may restrict the interaction of albumin with low-molecular-weight thiols.

Additionally, for each model, we calculated the average value of the number of water molecules in contact with the Sγ atom of Cys34 over the MD trajectories of all three replicas (we determined the number of water molecules within a radius of 3.5 Å from the sulfur atom of Cys34: the higher the degree of hydration of Cys34, the higher its accessibility for interaction with components of the aqueous environment, including potential donors/acceptors of the NO group). The results are presented in Table 1. According to the obtained values, the binding of OLA with unmodified albumin is accompanied by an increase in the average number of water molecules in contact with the sulfur atom of Cys34 by almost three times. This indicates a change in the local microenvironment and an increase in the availability of the thiol group for the solvent. S-nitrosylation resulted in a decrease in the number of water contacts; moreover, the nitrosylated forms HSA-NO and HSA-OLA-NO exhibited nearly identical numbers of water molecules contacting the Sγ atom of Cys34 regardless of OLA binding. The visual comparison of the Cys34 redox site (Figure 8) complements the hydration analysis (Table 1). Both approaches indicate that oleate binding and S-nitrosylation are associated with changes in the local environment of Cys34, with the HSA-OLA model showing the highest hydration of the Cys34 sulfur atom. These findings highlight the dependence of the local structural environment of the redox site on the ligand-binding and modification state of HSA.

A similar calculation was performed for the Oη atom of Tyr150 in site Sudlow I (a possible site of true esterase activity of albumin [24]), the Oη atom of Tyr411 in site Sudlow II (the most reactive site of pseudo-esterase activity of albumin [25]), the Nζ atom of Lys525 (the main glycation site of albumin [26]), and the side chain of Trp214 in site Sudlow I (the only tryptophan residue in the HSA molecule, the spectral properties of which largely determine the characteristics of the absorption and fluorescence spectra of the protein). In the case of Tyr150, as expected, in the presence of OLA, the average number of contacts with water molecules decreased by almost an order of magnitude compared to the HSA model, indicating a significant change in the local microenvironment of this residue and likely reflecting shielding of the site during OLA binding. Nitrosylation, however, had virtually no effect on the degree of hydration of this site. A similar trend is observed for Tyr411. In the case of Trp214, OLA binding also resulted in a reduction in the number of its contacts with water, and subsequent nitrosylation was accompanied by a partial restoration of hydration. The hydration of Lys525 remained virtually unchanged in all models studied, indicating preservation of the local environment and consistent with our previous computational experiments, which showed that fatty acid binding does not affect the interaction of glucose with Lys525 [27]. Thus, S-nitrosylation is accompanied by a selective change in the hydration of individual functionally significant regions of HSA.

It should be noted that Tyr150, Tyr411, and Lys525 are located within the major fatty acid-binding sites FA2, FA4, and FA5, respectively. Therefore, the observed site-specific hydration changes suggest that S-nitrosylation may modulate the local environment of FA-binding regions, potentially influencing the relative contribution of different fatty acid-binding sites to ligand distribution within the albumin molecule. This possibility may be particularly relevant under physiological conditions, where albumin is only partially saturated with fatty acids and the distribution of ligands among available binding sites is dynamic.

To explore whether the observed changes in the local environment of the Cys34 redox site may influence the spatial organization of potential transnitrosylation partners, we performed molecular docking of GSNO into the redox site of HSA and HSA-OLA models, as well as docking of GSH into the redox site of HSA-NO and HSA-OLA-NO models. Among the analyzed complexes, the HSA-OLA/GSNO pair demonstrated the most favorable spatial arrangement of the reactive groups, characterized by the shortest S–N distance among the generated docking poses. The detailed docking results, including predicted interaction energies, and subsequent short MD analysis of the obtained complexes are provided in the Supplementary Materials (Table S1 and Table S2 and Figure S1).

### 2.5. Investigation of HSA–GSNO Interaction by ^1^H NMR Spectroscopy

**Figure 9:**
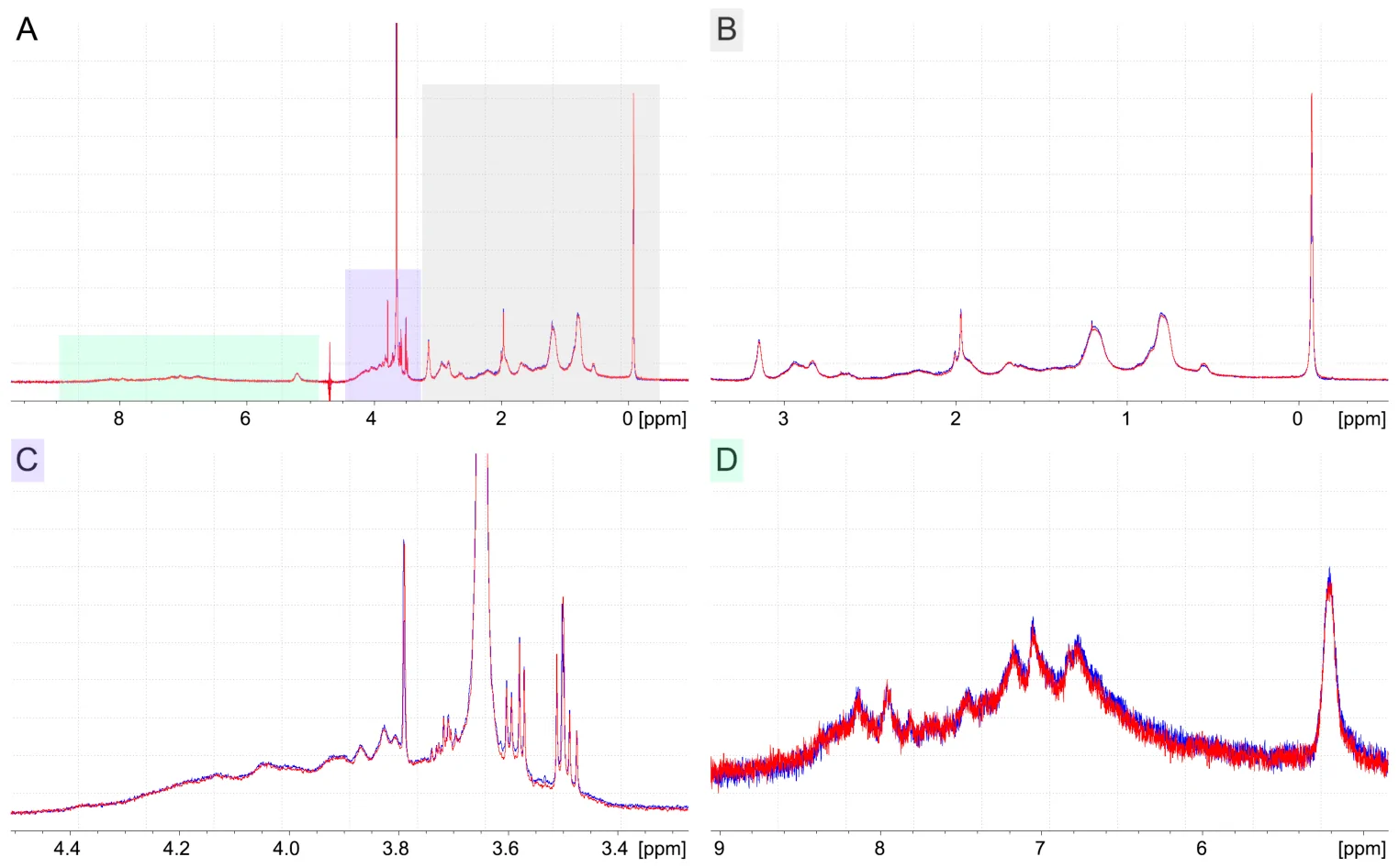
^1^H NMR spectra of HSA-37C (red) and HSA-GSNO-37C (blue): full spectra (**A**) and enlarged spectral regions at 3–0 ppm (**B**), 4.4–3.4 ppm (**C**), and 9.0–5.0 ppm (**D**). The shaded areas in Panel A correspond to the enlarged regions shown in Panels B (grey), C (purple), and D (green).

Since one of the objectives of this study was to evaluate possible structural changes in HSA upon S-nitrosylation, one-dimensional ^1^H NMR spectroscopy was used to analyze the conformational state of the protein. Spectra were recorded for native albumin (HSA) and a mixture of albumin with GSNO (HSA-GSNO), incubated at 37 °C (37C) or room temperature (25 °C, RT). Sample preparation is described in Section 4.2. Measurements at 37 °C were performed to assess the structural state of HSA under conditions close to physiological ones, whereas the spectra at 25 °C corresponded to standard NMR recording conditions. Figure 9 shows the ^1^H NMR spectra of HSA and HSA-GSNO incubated at 37 °C (HSA-37C and HSA-GSNO-37C samples). The spectra of the two samples were highly similar, with no noticeable differences between the corresponding spectral regions. A similar result was obtained for the HSA and HSA-GSNO samples maintained at room temperature (Figure S2).

Overall, the ^1^H NMR data did not reveal pronounced changes in the global conformational state of HSA upon incubation with GSNO. However, given the extensive signal overlap in the one-dimensional ^1^H NMR spectrum of HSA, these findings do not exclude localized structural rearrangements. It should be noted that a commercial HSA preparation not purified from endogenous fatty acids was used in the NMR experiments. Therefore, the spectra reflect both the conformational state of HSA and the presence of endogenous albumin-bound ligands. The presence of albumin-bound fatty acids was consistent with signals in the aliphatic region (2.5–0.8 ppm; Figure 9B and Figure S2B) and a signal at approximately 5.3 ppm (Figure 9D and Figure S2D), attributable to vinyl protons of unsaturated fatty acids [28]. The signal observed in the 0.0 to –0.2 ppm region (Figure 9A and Figure S2A) corresponds to the internal standard sodium trimethylsilylpropanesulfonate (DSS). Other signals were also observed in the spectra, but their unambiguous assignment will require additional NMR experiments, particularly two-dimensional NMR spectroscopy, to resolve overlapping signals in future studies.

The absence of substantial changes in the overall ^1^H NMR spectral profile indicates that S-nitrosylation does not induce large-scale unfolding or major rearrangement of the HSA structure. At the same time, considering the sensitivity of the NMR signal distribution to the global chemical environment of the protein, the observed spectral similarity is consistent with the MD results, which indicate preservation of the overall HSA fold accompanied by local rearrangements within functionally important regions.

## 3. Discussion

Human serum albumin is a multifunctional protein with binding, hydrolytic, and antioxidant activities. In addition, HSA can act as a ligand for certain enzymes and receptors, contributing to its involvement in various signaling pathways. These activities are mediated by specific functional sites of the protein, including fatty acid- and drug-binding sites, metal-binding sites, the redox-active Cys34 residue, nonenzymatic glycation sites, and regions involved in interactions with proteins and cellular receptors [1,6,27,29,30].

Despite the extensive characterization of most of these functional sites, much less is known about how structural changes in one site influence the properties of others. Such interconnections may play a key role in coordinating the diverse functions of HSA under physiological conditions. Therefore, the study of the molecular mechanisms underlying the relationship between the functional centers of HSA is of interest both from a fundamental point of view and for solving a wide range of biomedical problems. In the present study, we used computational modeling and ^1^H NMR spectroscopy to analyze the molecular mechanisms underlying the interplay between S-nitrosylation of the redox-active Cys34 site and long-chain fatty acid binding.

Using molecular modeling methods, we assessed how nitrosylation of FA-free and FA-loaded albumin affects its conformation, the local environment of the redox site, and the structural basis of HSA interaction with GSNO/GSH. Fatty acid binding is known to be accompanied by conformational changes in HSA, affecting the relative orientation of its domains and individual functional centers [31,32]. The results obtained in the present work complement these observations, showing that the influence of fatty acids is manifested not only in changes in the spatial organization of the protein but also in the features of its conformational dynamics and the local microenvironment of functionally significant regions. Such changes in conformational dynamics may influence the structural environment and adaptability of functional sites, potentially affecting the interactions of HSA with endogenous and exogenous ligands. The structural consequences of S-nitrosylation of HSA have been studied to a much lesser extent. According to our data, nitrosylation does not induce major rearrangements of the global protein fold, but it alters the local microenvironment of a number of functionally significant regions. These observations support the concept that modifications at one functional region of HSA may influence distant ligand-binding sites through the interconnected nature of the protein conformational landscape. For example, in vitro experiments have shown that Cys34 oxidation is accompanied by a change in albumin affinity for site III ligands, while the effect on sites I and II is significantly weaker [33,34,35]. However, in vivo studies indicate that in diseases accompanied by the accumulation of oxidized forms of albumin, the binding capacity of both site I and site II is reduced [36,37]. One possible explanation for these differences is that, under physiological conditions, the structural state of HSA is influenced not only by redox modifications of Cys34 but also by interactions with endogenous ligands, primarily fatty acids. Thus, endogenous fatty acids may act as important modulators of the conformational landscape of HSA, influencing how local modifications are manifested at the level of the overall protein conformation.

Regarding the ^1^H NMR spectroscopy results, the high similarity between the one-dimensional NMR spectra of native HSA and HSA after incubation with GSNO indicates that S-nitrosylation does not induce substantial changes in the global conformational state of the protein detectable by this method. However, due to the extensive signal overlap characteristic of the ^1^H NMR spectrum of HSA, this approach does not allow detailed characterization of local changes in the microenvironment of individual amino acid residues. Therefore, the absence of pronounced spectral differences does not exclude possible rearrangements in the Cys34 region. The preservation of the overall HSA fold observed by ^1^H NMR is consistent with the molecular dynamics results, which indicated that S-nitrosylation of fatty acid-loaded HSA does not cause large-scale rearrangement of the protein conformational landscape but may modulate the distribution and flexibility of conformational states, as reflected by the slight elongation of the PCA-based conformational ensemble. Thus, the NMR and MD approaches provide complementary structural information, with NMR reflecting the preservation of the global fold and MD revealing localized alterations in conformational dynamics and hydration patterns within functionally important regions.

The obtained results further emphasize the importance of analyzing protein structural dynamics for understanding the mechanisms underlying biological function. Although gene expression determines the amount of synthesized protein, its functional state is shaped by multiple subsequent regulatory mechanisms, including post-translational modifications [38,39] and interactions with endogenous ligands [40]. These processes can alter the conformational ensemble of a protein, its dynamic properties, and its ability to interact with other molecules [41,42]. In the case of HSA, fatty acid binding and redox modification of Cys34 represent two interconnected regulatory processes that may contribute to shaping the functional state of the protein without altering its amino acid sequence.

In this context, the results obtained in the present study are consistent with the hypothesis that fatty acids, when bound to albumin, simultaneously regulate two of its functions—transport and antioxidant activity—acting as a “mediator” of the influence of one activity of albumin on another [32]. In a broader context, fatty acids may be considered not primarily as factors affecting the efficiency of albumin nitrosylation, but rather as modulators of HSA involvement in the NO transport and distribution system. NO-dependent effects may be determined not only by the concentration of S-nitrosylated compounds but also by the balance between their formation and breakdown. Therefore, fatty acids may potentially contribute to the redistribution of NO groups between different thiol-containing pools. It is known that plasma free fatty acid levels vary significantly depending on the physiological and pathological state of the body. Increased concentrations of non-esterified fatty acids are observed in metabolic syndrome, diabetes mellitus, obesity, inflammatory conditions, and certain diseases [43]. Under these conditions, albumin is highly saturated with fatty acids, which could potentially alter the nature of NO signaling and potentially influence NO group distribution within the vascular bed and tissues. From a practical perspective, these data may be important for interpreting the results of studies that use S-nitrosylated albumin levels as a biomarker of NO-dependent processes. Without taking into account the degree of albumin saturation with fatty acids, an assessment of the functional significance of HSA-NO may be incomplete. This is especially relevant for research in cardiovascular and metabolic diseases, in which changes in the lipid profile and NO signaling are closely interrelated.

These considerations are also important in the context of pharmacological studies involving albumin. The protein binds many drugs and toxic substances, making a significant contribution to their pharmacokinetics and toxicokinetics. When developing new drugs, analysis of their interactions with albumin is essential [44,45,46]. Albumin-based drug delivery systems are widely used [47,48], and increasing attention is being focused on the development of HSA variants with tailored functional properties [49,50]. Within this context, an important methodological issue in determining the binding constants of drug compounds to HSA is the choice of albumin preparation. A recent comparative study of ten commercial HSA preparations, including fatty acid-free and recombinant HSA, revealed significant differences in the fatty acid content and ligand-binding properties of these samples [51]. Moreover, the studied recombinant HSA expressed in rice contained a significant amount of free fatty acids, which shows that recombinant origin in itself does not guarantee the absence of endogenous ligands. The authors emphasized the need for careful characterization and selection of HSA drugs in ligand-protein interaction studies. On the one hand, using an HSA purified from endogenous ligands allows evaluation of the protein’s intrinsic properties and the effect of a specific modification. On the other hand, for assessing the potential behavior of a drug in the body, HSA containing endogenous fatty acids may be a more physiologically relevant model, as this is the state in which albumin circulates in plasma. Thus, commercial HSA without additional removal of endogenous fatty acids can also be considered as a physiologically relevant model for studying drug-albumin interactions. Our research, involving albumin that had not been stripped of fatty acids, unexpectedly received support in terms of the technological methods or know-how used to produce recombinant albumin: addition of oleic acid to the medium is likely to play an important role, in cooperation with gene manipulation, in achieving high expression levels of recombinant human serum albumin (rHSA) for the purpose of commercial production. A small amount of oleic acid (0.01% (*w*/*v*)) doubled the rHSA production level [52].

Understanding these molecular mechanisms and modifications is also crucial for clinical applications, given the multitude of functions this protein performs during critical physiological states. Synthetic colloid solutions were previously considered at least as effective as natural colloids, as well as being cheaper and easily available. Currently, albumin seems to be safer for people than synthetic colloids, according to numerous studies [53,54,55]. Crystalloids have small molecules, are cheap, are easy to use, and provide immediate fluid resuscitation but may increase oedema. Colloids have larger molecules, cost more, and may provide swifter volume expansion in the intravascular space; on the other hand, sometimes they induce allergic reactions, blood clotting disorders, and kidney failure [56,57]. The mechanisms of negative or positive reactions caused by albumin are poorly understood, and there are several studies that reveal intermolecular effects. For example, the crowded environment in combination with serum albumin could influence whether lipoprotein lipase (LPL) undergoes irreversible inactivation and aggregation or reversible LPL oligomer formation [58].

Our recent work substantiates and consistently advances the concept of the integrative role of albumin, which acts as a unique biochemical interface that both reflects the body’s physiological state and largely determines it through interactions with numerous enzymes and receptors [1,27,29,30,59]. The nature of this interaction obviously depends to a significant extent on the numerous modifications of albumin—whether in pooled plasma or purified from plasma. This highlights the need to produce recombinant albumin with standardized amounts and types of these modifications. The transition from plasma-derived to rHSA represents a pivotal advancement in ensuring a safe and sustainable supply of this essential therapeutic protein [60,61,62]. In the future, it appears promising to obtain a modified recombinant albumin that possesses transport, enzymatic and antioxidant functions but is incapable of interacting with functionally significant receptors and enzymes (receptor for advanced glycation end-products, angiotensin-converting enzyme, CD36 receptor, TGF-β receptor, etc.). Factors influencing the level of albumin gene expression in different expression platforms warrant further study.

Taken together, both our fundamental findings and the discussed translational challenges emphasize the need for further investigation of the interplay between different functional sites of HSA. Albumin, with its broad range of physiological functions and well-characterized three-dimensional structure, serves as a convenient model for studying general principles of protein structure–function relationships and their targeted engineering for biomedical applications.

## 4. Methods

### 4.1. Molecular Modeling

For molecular docking of GSH and GSNO into the redox site of albumin, the HSA, HSA-OLA, HSA-NO and HSA-OLA-NO models were generated from the crystal structures 1AO6 and 1GNI following the processing procedure described in Section 2.1, using the CHARMM-GUI web server (Lehigh University, Bethlehem, PA, USA) [23] and subsequent energy minimization using software GROMACS 2023.3 (Stockholm University, Stockholm, Sweden) [63]. Crystal structures of GSH and GSNO from the Cambridge Crystallographic Data Center [64] were used as three-dimensional models of the ligands. The docking procedure was performed using the Autodock Vina v1.1.2 program (The Scripps Research Institute, La Jolla, CA, USA) [65]. In the studied binding site, a search area of 20 × 20 × 20 Å was set. The number of runs (exhaustiveness) was taken to be 20. The allowable maximum scatter of conformational energies in the output file (energy_range) was taken to be 3 kcal/mol. The number of the most optimal (energetically favorable) conformations in the output file (num_modes) was set to ten. The protein molecule was set to be rigid, and the conformation of the ligands could vary.

Conformational changes in the studied HSA, HSA-OLA, HSA-NO and HSA-OLA-NO models and their complexes with GSH and GSNO were calculated by the MD simulation using the software GROMACS 2023.3 (Stockholm University, Stockholm, Sweden) [63] in the CHARMM36 force field [66]. The models were prepared for the simulation with the help of the CHARMM-GUI web server [23]. The molecules were virtually placed in a periodic cubic cell filled with water molecules. The distance between the protein surface and the cell boundary was 1 nm. To neutralize the charge, sodium ions were added to the system. The TIP3P water model (transferable inter-molecular potential with 3 points) was used to describe water molecules [67].

Before production of MD simulations, all systems were subjected to energy minimization using the steepest-descent algorithm (5000 steps; convergence criterion of 1000 kJ mol^−1^ nm^−1^). Positional restraints were applied to the solute during this step, with force constants of 400 and 40 kJ mol^−1^ nm^−2^ for the protein backbone and side-chain atoms, respectively. The minimized systems were then equilibrated for 1 ns in the NVT ensemble at 303.15 K using the V-rescale thermostat [68], with a temperature-coupling time constant of 1 ps. The integration time step was 1 fs.

Production of MD simulations was performed in the NPT ensemble at 303.15 K and 1 bar. Temperature and pressure were kept constant using the V-rescale thermostat [68] and C-rescale barostat [69] with coupling time constants of 1 and 5 ps, respectively. Long-range electrostatic interactions were treated by the particle-mesh Ewald method [70]. Lennard-Jones interactions were force-switched off between 1.0 and 1.2 nm. The LINCS algorithm (linear constraint solver for molecular simulations) was used to constrain bond length [71]. The time step for MD simulations was set to 0.002 ps. For the HSA, HSA-OLA, HSA-NO, and HSA-OLA-NO models, three independent production MD simulations were performed, each with a duration of 100 ns. Single 20 ns MD simulations were carried out for the HSA-GSH/GSNO complexes.

Oleic acid topology and parameters were generated using the CHARMM-GUI web server within the CHARMM36 force field framework, with the molecule represented in its deprotonated form (oleate, net charge −1). Parameters for GSH and GSNO were obtained using the CHARMM-GUI ligand parameterization module based on the docked conformations of these molecules. Both ligands were assigned a net charge of −1.

The MD trajectories were analyzed using standard GROMACS tools. RMSD, RMSF, Rg, and SASA were calculated using the GROMACS commands gmx rms, gmx rmsf, gmx gyrate, and gmx sasa, respectively, for the entire 100 ns trajectories. Conformational clustering was performed using the GROMACS gmx cluster command with an RMSD cutoff of 0.15 nm. Clustering analysis was performed for the equilibrated part of each trajectory (50–100 ns). Principal component analysis (PCA) was performed using the GROMACS gmx covar and gmx anaeig commands based on Cα atoms of the protein. For systems with three independent replicas, PCA was performed on a combined trajectory generated from all replicas to characterize the overall conformational landscape and compare the dominant collective motions between different albumin states.

### 4.2. NMR Spectroscopy

On the day of the experiment, 52.5 mg of non-defatted HSA (IMTEK, Moscow, Russia) was dissolved in 2.5 mL of 10 mM Tris buffer (pH 7.4), yielding a final protein concentration of 316 μM. The synthesis of GSNO was carried out on the day of the experiment according to the method described in [72]. Reduced glutathione (GSH, 18 mg; Lenreactiv, St. Petersburg, Russia) was dissolved in 1 mL of 10 mM Tris buffer (pH 7.4), and 4.14 mg of sodium nitrite (Lenreactiv, St. Petersburg, Russia) was dissolved in 1 mL of the same buffer. The GSH and sodium nitrite solutions were then mixed, and hydrochloric acid was added dropwise until an intense pink color developed. The reaction mixture was subsequently incubated for 20 min at room temperature in the dark. The calculated concentration of the resulting GSNO solution was 29.3 mM. Then, control and experimental samples were prepared, as described in Table 2. The prepared samples were scanned by ^1^H NMR in a Bruker Avance III 500 NMR spectrometer (Bruker BioSpin, Rheinstetten, Germany) at room temperature (25 °C). To suppress the water signal, the excitation sculpting method was used [73]. At each time point, 128 scans were performed. DSS (0.0–−0.2 ppm) was used as the internal standard. Chemical shifts δ of solution components accurate to 0.001 ppm were calibrated against tetramethylsilane Si(CH_3_)_4_.

## 5. Limitations of the Study

The presented study has several limitations. The molecular dynamics method relies on approximate force fields, which can impact the accuracy of reproducing protein conformational dynamics. Moreover, the duration of the MD trajectories (100 ns) may be insufficient to fully analyze large-scale structural changes in HSA. However, in combination with three independent replicates for each system, the chosen simulation duration allows us to evaluate reproducible features of the structural stability and conformational behavior of the protein under different conditions. After an initial period of adaptation of the starting structure, the RMSD values stabilized (Figure 2), indicating that the stable conformation of HSA was maintained throughout the studied trajectories. Increasing the simulation duration to the microsecond range will help to evaluate slow conformational transitions of HSA that are not visible within 100 ns trajectories.

The use of one-dimensional ^1^H NMR spectroscopy for studying a large protein such as HSA has inherent limitations due to extensive signal overlap, which restricts detailed analysis of structural changes at the level of individual amino acid residues. Therefore, the obtained spectral data should be considered primarily as information on the global conformational state of the protein rather than as a tool for mapping local structural rearrangements. More detailed characterization of local structural changes could be achieved using two-dimensional NMR approaches (including isotopically labeled HSA samples), as well as methods providing information on conformational rearrangements of individual protein regions, such as hydrogen–deuterium exchange coupled to mass spectrometry (HDX-MS) or chemical cross-linking followed by mass spectrometry (XL-MS). Other biophysical approaches, including circular dichroism spectroscopy and small-angle X-ray scattering (SAXS), could provide additional information on overall conformational changes. It should also be noted that the GSNO sample used in this study was synthesized immediately before the experiment according to a published protocol. Additional verification of the chemical purity of the obtained sample using independent analytical approaches was not performed; therefore, the possible presence of synthesis byproducts or partial degradation of GSNO and HSA-NO during sample preparation and incubation cannot be completely excluded. Further experimental validation using more sensitive structural and biophysical approaches is required to directly characterize local conformational rearrangements induced by S-nitrosylation and determine the kinetics and molecular determinants of HSA interactions with low-molecular-weight nitrosothiols.

Furthermore, both in silico molecular modeling and in vitro experiments cannot fully reproduce the complex microenvironment of albumin in vivo. In the calculations, HSA bound to seven oleic acid molecules was used as a model, whereas under physiological conditions, the degree of albumin saturation with fatty acids varies widely and depends on the metabolic state of the organism [74,75], and different fatty acids can affect protein structure differently [22]. Therefore, further study of the influence of other physiologically significant fatty acids and different degrees of albumin loading, as well as the combined effects of nitrosylation, glycation, oxidation and fatty acid binding, is of considerable interest for a more complete understanding of the structural mechanisms that determine the functional properties of HSA.

## 6. Conclusions

In the present study, the structural mechanisms underlying the interplay between fatty acid binding and Cys34 S-nitrosylation in human serum albumin were investigated using computational modeling and NMR spectroscopy. According to the in silico data, OLA binding is accompanied by changes in the conformational dynamics of albumin, alterations in the local microenvironment of Cys34, and modifications of other functionally significant regions of the protein. These changes may influence the structural environment of the Cys34 thiol group and the interaction of albumin with low-molecular-weight S-nitrosothiols. Regarding Cys34 S-nitrosylation, molecular dynamics results indicate that this modification induces local changes in the microenvironment of functional sites and affects the distribution of accessible conformational states of the protein in a fatty acid-dependent manner but does not result in pronounced rearrangement of the global HSA structure. The latter is consistent with the ^1^H NMR data demonstrating preservation of the overall conformational state of the protein after interaction with GSNO. Thus, the obtained data indicate that the functional state of HSA is determined not only by the properties of individual regulatory sites but also by their interconnection within the unified dynamic protein structure, highlighting the important role of endogenous ligands and post-translational modifications in the regulation of its functional properties. The presented approach emphasizes the importance of considering protein conformational dynamics and the influence of physiological ligands when studying the mechanisms that determine protein functional states.

## Figures and Tables

**Table 1:** Average number of water molecules within a 3.5 Å radius of HSA functional sites depending on the albumin state. For each model, averaging was performed over all trajectories of all three MD replicas.

	HSA	HSA-NO	HSA-OLA	HSA-OLA-NO
atom Sγ of Cys34 (DIA)	0.8	0.5	2.1	0.5
atom Oη of Tyr150 (DIIA)	3.1	3.4	0.4	0.1
sidechain of Trp214 (DIIA)	23.2	22.6	17.3	19.6
atom Oη of Tyr411 (DIIIA)	6.9	5.4	1.7	1.8
atom Nζ of Lys525 (DIIIB)	8.4	7.1	7.9	8.1

**Table 2:** Preparation of control and experimental samples for NMR spectroscopy.

Sample	Sample Preparation
HSA-RT	530 μL of HSA solution was mixed with 70 μL of Tris buffer, 60 μL of D_2_O, and 30 μL of DSS (final HSA concentration: 242 μM). The sample was incubated for 30 min at room temperature (25 °C) and then centrifuged three times using 10 kDa centrifugal filters at 12,000× *g* for 10 min each.
HSA-37C	530 μL of HSA solution was mixed with 70 μL of Tris buffer, 60 μL of D_2_O, and 30 μL of DSS (final HSA concentration: 242 μM). The sample was incubated for 30 min at 37 °C in a BioSan thermostatic incubator (BioSan, Riga, Latvia) and subsequently centrifuged three times using 10 kDa centrifugal filters at 12,000× *g* for 10 min each.
HSA-GSNO-RT	530 μL of HSA solution was mixed with 70 μL of GSNO solution, 60 μL of D_2_O, and 30 μL of DSS (final concentrations: GSNO, 2.97 mM; HSA, 242 μM; GSNO/HSA molar ratio = 12). The sample was incubated for 30 min at room temperature (25 °C) and subsequently centrifuged three times using 10 kDa centrifugal filters at 12,000× *g* for 10 min each.
HSA-GSNO-37C	530 μL of HSA solution was mixed with 70 μL of GSNO solution, 60 μL of D_2_O, and 30 μL of DSS (final concentrations: GSNO, 2.97 mM; HSA, 242 μM; GSNO/HSA molar ratio = 12). The sample was incubated for 30 min at 37 °C in a BioSan thermostatic incubator and subsequently centrifuged three times using 10 kDa centrifugal filters at 12,000× *g* for 10 min each.

## Data Availability

The data presented in this study are available from the corresponding authors upon reasonable request.

## Supplementary Materials

The following supporting information can be downloaded at: https://www.mdpi.com/article/10.3390/ijms27219687/s1.

## Abbreviations

The following abbreviations are used in this manuscript:

distS-N	Distance between the nitrogen atom of the NO group of GSNO or HSA-NO and the sulfur atom of the thiol group of GSH or HSA
FA	Fatty acid
GSH	Glutathione
GSNO	S-nitrosoglutathione
HSA	Human serum albumin
HSA-NO	S-nitrosoalbumin
HSA-OLA	Complex of HSA with 7 molecules of oleic acid
HSA-OLA-NO	Complex of HSA-NO with 7 molecules of oleic acid
LPL	Lipoprotein lipase
MD	Molecular dynamics
NO	Nitric oxide
OLA	Oleic acid
PCA	Principal component analysis
Rg	Radius of gyration
RMSD	Root mean square deviations
RMSF	Root mean square fluctuations
SASA	Solvent accessible surface area
