# Supplementary material for: Structural Determinants Underlying the Interplay Between Fatty Acid Binding and S-Nitrosylation of Human Serum Albumin
Source: Int J Mol Sci. 2026 Oct 29;27(21):9687. doi: 10.3390/ijms27219687 (PMC13762914; doi:10.3390/ijms27219687)
Supplement: Supplementary file 1 [file ijms-27-09687-s001.zip › ijms-4527577-supplementary.pdf]

Molecular docking of GSH and GSNO into the redox site of various modifications of HSA

The result of each docking run was a set of ten predicted binding poses ranked according to the docking score, with the most favorable conformations corresponding to the lowest (most negative) predicted binding energy values (ΔG). For analysis, from the resulting set of 10 energetically favorable conformations, the conformation with the minimum distance between the nitrogen atom of the NO group of GSNO or HSA-NO and the sulfur atom of the thiol group of GSH or HSA (distS-N) was selected. This criterion was chosen because a shorter distance between the sulfur and nitrogen atoms of the reactive groups indicates a more favorable spatial arrangement for a potential transnitrosylation reaction. The result is presented in Figure S1 and Table S1.

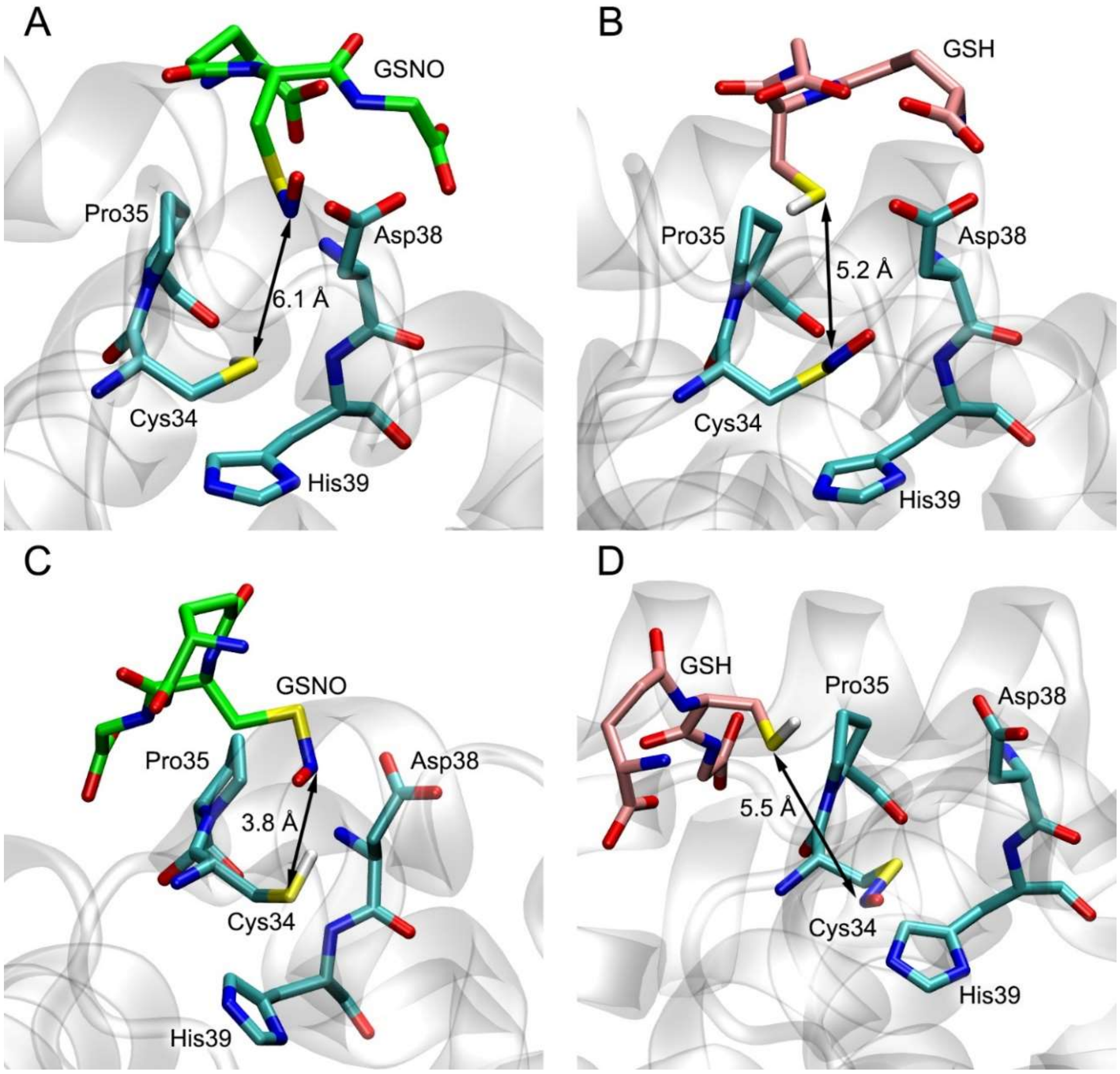

Figure S1. The result of molecular docking of GSH and GSNO into the redox sites of various modifications of the albumin molecule: HSA (A), HSA-NO (B), HSA-OLA (C) and HSA-OLA-NO (D). GSH, GSNO, and redox site amino acids are represented as sticks, with the protein backbone represented by a gray ribbon. The carbon atoms of GSH and GSNO are highlighted in pink and green, respectively. Hydrogen atoms are not shown for clarity of the figure, except for the hydrogen of the thiol groups Cys34 and GSH.

Table S1. Result of molecular docking of GSH and GSNO into the redox site of various modifications of albumin

receptor / ligand	ΔG, kcal/mol	distS-N, Å
HSA / GSNO	–2.1	6.1
HSA-NO / GSH	–2.9	5.2
HSA-OLA / GSNO	–3.8	3.8
HSA-OLA-NO / GSH	–3.7	5.5

ΔG – estimated binding free energy; distS-N – distance between the nitrogen atom of the NO group GSNO or HSA-NO and the sulfur atom of the thiol group HSA or GSH

Among the analyzed complexes, the HSA-OLA-GSNO pair demonstrated a binding mode with geometric parameters most favorable for a potential transnitrosylation reaction (Figure S1C). Moreover, the predicted binding energy score is also the most negative for this pair (Table S1). The ΔG value for the HSA-OLA-NO complex with GSH is also quite low (Table S1), but the distance between the reacting groups is higher (Figure S1D) than in the case of the HSA-OLA complex with GSNO. The opposite result was obtained for FA-free albumin. Firstly, the predicted binding scores suggest weaker ligand-protein interactions (Table S1) than in the case of OLA-loaded forms of HSA. Secondly, the ΔG and distS-N parameters for the HSA-NO interactions with GSH (Figure S1B) and HSA with GSNO (Figure S1A) are generally comparable, suggesting similar predicted interaction modes and comparable spatial arrangements of the reactive groups.

To further evaluate the stability of the docking-derived binding modes, the obtained HSA-GSH/GSNO complexes were subjected to short single-trajectory MD simulations. After energy minimization, the systems were equilibrated for 1 ns under the NVT ensemble and subsequently simulated for 20 ns. The distance between the sulfur atom of the thiol group and the nitrogen atom of the NO group (distS-N) was monitored throughout the simulations. Table S2 summarizes the distS-N values obtained for the initial docking poses, minimized structures, NVT-equilibrated structures, and mean values calculated over different MD trajectory intervals. The duration of the initial period during which the S-N distance remained below 5 Å was calculated as an indicator of the preservation of a potentially productive transnitrosylation geometry. The analysis demonstrated that the docking-derived geometries were generally maintained after energy minimization and NVT equilibration. However, during the subsequent MD simulations, all complexes exhibited a gradual increase in the distS-N value, indicating loss of the initial reactive orientation. Among the investigated systems, the HSA-OLA-GSNO complex showed the slowest initial increase in the S-N distance. Although the predicted GSNO–albumin interaction is relatively weak (ΔG ≈ –3.8 kcal/mol), the HSA-OLA-GSNO complex was the only system that retained a potentially productive geometry (distS-N ≤ 5 Å) during the first 100 ps of the MD trajectory (Table S2). These results suggest that oleate binding may facilitate transient positioning of GSNO near

the Cys34 thiol group, increasing the probability of productive encounters rather than forming a permanently stabilized complex. A more detailed characterization of HSA-GSH/GSNO interactions would require longer MD simulations with multiple independent trajectories, and analysis of a broader conformational ensemble.

Table S2. Evolution of the S-N distance (distS-N, Å) during molecular dynamics refinement of docking-derived GSH/GSNO complexes with different HSA forms.

**Complex**	**HSA-GSNO**	**HSA-NO-GSH**	**HSA-OLA-GSNO**	**HSA-OLA-NO-GSH**
Docking pose	6.1	5.2	3.8	5.5
Minimized structure	6.2	5.8	4.0	5.5
NVT-equilibrated structure (1 ns)	6.1	8.3	4.2	5.6
mean MD (0-0.5 ns)	6.8	10.6	5.9	8.9
mean MD (0-1 ns)	6.9	11.7	7.1	8.6
mean MD (0-2 ns)	8.2	15.6	9.4	8.8
mean MD (0-5 ns)	19.9	33.6	12.1	13.5
mean MD (0-10 ns)	32.5	44.0	12.7	18.8
mean MD (0-20 ns)	45.6	37.2	29.1	41.0
Duration of distS-N < 5 Å (ps)	0	0	100	0

Investigation of HSA–GSNO interaction by 1H NMR spectroscopy

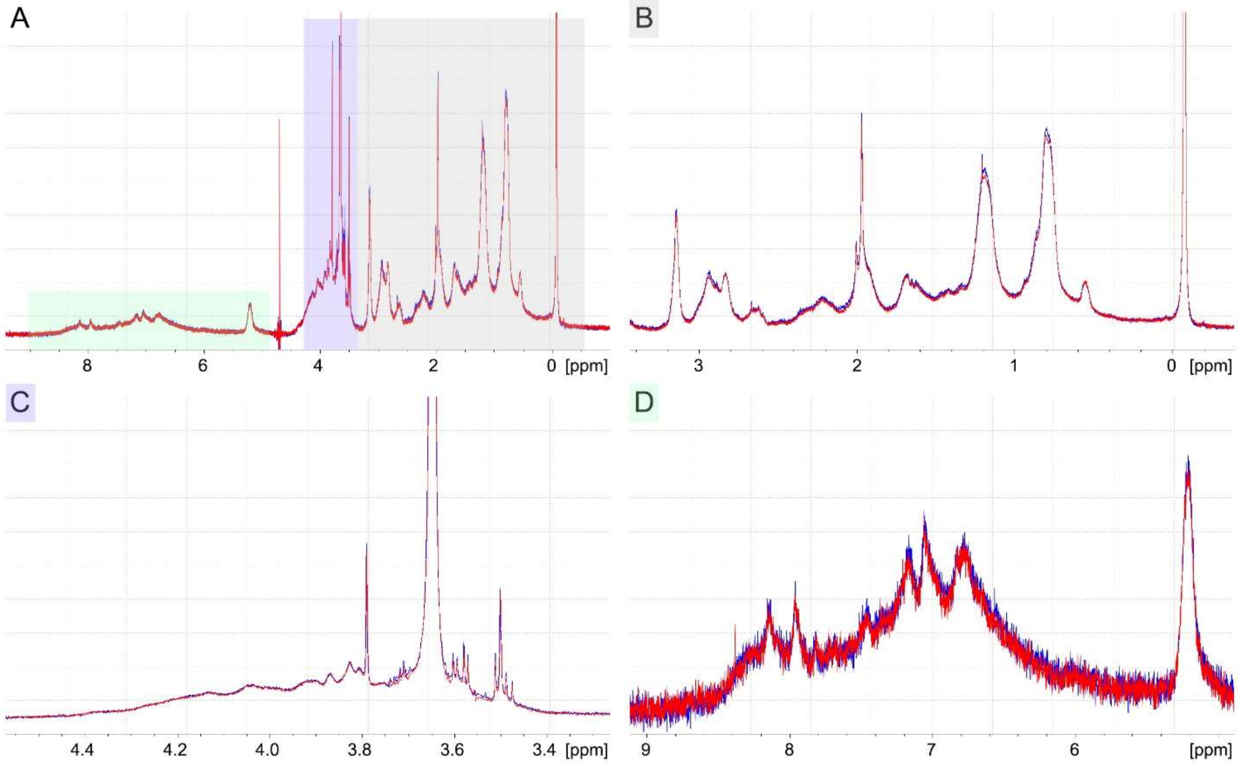

Figure S2. ^{1}H NMR spectra of HSA-RT (red) and HSA-GSNO-RT (blue): full spectra (A) and enlarged spectral regions at 3–0 ppm (B), 4.4–3.4 ppm (C), and 9.0–5.0 ppm (D). The shaded areas in Panel A correspond to the enlarged regions shown in Panels B (grey), C (purple), and D (green).
